# Protective Effects of *Agrimonia pilosa* Ledeb. In Myocardial Fibrosis: Inhibition of Mitophagy Mediated by the FOXO Signaling Pathway

**DOI:** 10.1002/fsn3.71778

**Published:** 2026-04-15

**Authors:** Jian Chen, Zhixiang Wei, Jiaqi An, Dantong Li, Ying Gu, Yixin Zhang, Muqing Zhang

**Affiliations:** ^1^ Hebei University of Chinese Medicine Shijiazhuang China; ^2^ Hebei International Joint Research Center of Chinese Medicine Resource Utilization and Quality Evaluation Shijiazhuang China; ^3^ The Fourth Hospital of Hebei Medical University Shijiazhuang China

**Keywords:** *Agrimonia pilosa* Ledeb, apoptosis, FOXO signaling pathway, functional food, inflammatory response, mitophagy, myocardial fibrosis, oxidative stress

## Abstract

*Agrimonia pilosa* Ledeb. (APL) is an edible and medicinal plant, which has a favorable cardioprotective effect. Myocardial fibrosis (MF) is a hallmark pathological feature of various cardiovascular diseases. This study aims to evaluate its protective effects against isoproterenol (ISO)‐induced MF and investigate the underlying mechanisms. HPLC was employed to analyze the main active ingredients in APL. Network pharmacology methods, combined with experimental validation, elucidated the mechanism by which APL modulates mitophagy to alleviate MF. HPLC analysis showed that six ingredients were identified. We demonstrated that APL significantly attenuated myocardial injury, enhanced cardiac function, inhibited oxidative stress and apoptosis, and effectively ameliorated MF progression. Network pharmacological predictions and in vivo experimental validation demonstrated that APL exerts its therapeutic effects through regulation of the FOXO signaling pathway and suppression of excessive mitophagy. Furthermore, we artificially elevated FOXO1 expression in vitro, which reversed the effects of APL, as evidenced by increased expression of mitophagy and fibrosis‐related proteins. Consistent results from both in vivo and in vitro experiments demonstrated that APL attenuates ISO‐induced MF and suppresses mitophagy mediated by the FOXO signaling pathway.

## Graphical abstract

*Agrimonia pilosa* Ledeb. has the effect of repairing myocardial injury, reducing myocardial hypertrophy, and improving MF. Mechanistically, it acts by inhibiting mitophagy mediated by the FOXO signaling pathway, as validated via network pharmacology, in vitro*,* and in vivo experiments.

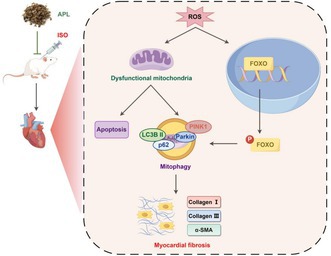

## 1 Introduction

*Agrimonia pilosa Ledeb*. (APL), a plant with both medicinal and edible applications, is considered a high‐quality wild vegetable and is traditionally consumed with red dates to reduce fatigue and exhaustion. As an herbal medicine, APL demonstrates anti‐inflammatory and antioxidant properties (Feng et al. 2021; Kim et al. 2020; Liu et al. 2014; Trinh et al. 2022). Flavonoids derived from APL have been documented to exhibit potent antioxidant properties, including free‐radical scavenging and protection against oxidative DNA damage (Zhu et al. 2017). Our previous study found that APL reduced oxidative stress and apoptosis and ameliorated ISO‐induced acute myocardial infarction (AMI) (Zhang, Chen, et al. 2022). However, it remains unclear whether APL exerts similar protective effects against other cardiovascular diseases.

Myocardial fibrosis (MF) is a common pathological basis for various cardiovascular diseases, characterized by the excessive proliferation of cardiac fibroblasts, abnormal deposition and irregular distribution of collagen, alongside aberrant inflammatory responses, as well as cardiomyocyte necrosis and apoptosis (Yazğan et al. 2025; Yıldızhan et al. 2026). According to worldwide statistics, the annual incidence of MF is as high as 1.7%, and it is a universal pathological link in various myocardial diseases including hypertension, myocardial infarction, and heart failure (Zhao et al. 2020). There is no method to completely reverse MF, and angiotensin‐converting enzyme inhibitors, β‐adrenergic receptor, and mineralocorticoid‐receptor antagonists are utilized in clinical practice for anti‐fibrosis (López et al. 2021). However, these drugs may produce a range of side effects, including kidney impairment and hypotension (Fang et al. 2024). Natural products can retard the progress of MF and are generally safe and effective (Zhang et al. 2024). Therefore, there is an urgent need to develop effective, low‐toxicity, plant‐based interventions.

Mitophagy selectively targets and degrades dysfunctional mitochondria through autophagy, which is imperative for maintaining the overall physiological function of the organism (Lu et al. 2023). The putative kinase 1 (PINK1)/Parkin pathway is a central regulatory mechanism in mitophagy, orchestrating the initiation, signaling, and selective degradation of mitochondria, thereby maintaining cellular energy metabolism (Wang, Long, et al. 2023). PINK1, a highly conserved mitochondrial protein, is integral to the modulation of mitochondrial function. Parkin mediates mitochondrial substrate ubiquitination and induces mitophagy (Li et al. 2023). When mitochondria are damaged, PINK1 levels are observably elevated at the outer mitochondrial membrane, facilitating the recruitment of Parkin and activating mitophagy (Jayatunga et al. 2020). Excessive mitophagy results in the removal of large numbers of mitochondria, which disrupts the metabolic processes within mitochondria. This process is accompanied by the release of excess reactive oxygen species (ROS) and inflammatory cytokines, contributing to mitochondrial damage and apoptosis, creating a vicious cycle that ultimately leads to fibrosis (Yang et al. 2023).

Forkhead box class O family (FOXOs) comprises highly conserved transcription factors that significantly contribute to maintaining cellular homeostasis (Cheng 2019). FOXO1 and FOXO3 regulate mitophagy, oxidative stress, and apoptosis (Sanchez et al. 2014). This regulation is mediated by various post‐translational modifications, including phosphorylation of the FOXO1A and FOXO3A proteins (Bagam et al. 2021). Recent studies suggested that mitophagy orchestrated by the FOXO signaling pathway has been established in diseases such as diabetic cardiomyopathy and pulmonary fibrosis (Singh et al. 2023; Zhang et al. 2021). However, the regulatory effects of the FOXO signaling pathway on mitophagy in isoproterenol (ISO)‐induced MF remain to be fully elucidated.

The potential therapeutic effectiveness of APL for MF has not been determined. Therefore, we analyzed the primary ingredients of APL using High Performance Liquid Chromatography (HPLC). We screened the potential mechanism of APL to improve MF using network pharmacology, and then preliminarily verified the effect of APL on the FOXO signaling pathway and mitophagy in MF mice and H9c2 cells.

## 2 Materials and Methods

### 2.1 Chemicals and Reagents

APL (24040138, Sinopharm Group Le‐Ren‐Tang Medicines Co. Ltd., Hebei, China); ISO (I0260‐5, TCI, Shanghai, China); propranolol (PRO, H32020267, Yunyang Phamaceutical Group Co. Ltd., Jiangsu, China); Isoflurane (2024081901, Jinan Ante Biochemical Pharmaceutical, Shandong, China); Methanol, Acetonitrile, Formic acid (F24OAN205, F24O4N202, 244337, Fisher Chemical, Pittsburgh, USA); Gallic acid, Rutin, Ellagic acid, Taxifolin, Quercetin, and Kaempferol (CAS: 149‐91‐7, 153‐18‐4, 476‐66‐4, 480‐18‐2, 117‐39‐5, 520‐18‐3, Desite, Sichuan, China); Creatine Kinase (CK), CK MB isoenzyme (CK‐MB), L‐Lactate Dehydrogenase (LDH) Test Reagent Kit (20240430, 20240604, 20240912, DERUI BIOLOGY, Guangzhou, China); superoxide dismutase (SOD), Malondialdehyde (MDA), Glutathione peroxidase (GSH‐Px), Catalase (CAT) Test Reagent Kit (G4306, G4302, G4310, G4307, Servicebio, Hubei, China); Tumor necrosis factor‐α (TNF), interleukin (IL)‐6, IL‐1β ELISA Kit (GEM0004, GEM0001, GEM0002, Servicebio, Hubei, China); Procollagen Type I C‐Terminal Propeptide (PICP) and carboxyterminal propeptide of type I procollagen (ICTP) (JL10121‐48t, JL20125‐48T, Jianglai Biotechnology, Shanghai, China); GAPDH (380626, ZEN BIO, Sichuan, China); FOXO1A, p‐FOXO1A, FOXO3A, p‐FOXO3A, PINK1, Parkin, Beclin‐1, Sequestosome 1 (p62), microtubule‐associated protein 1 light chain 3 alpha B (LC3B), B‐cell lymphoma‐2 (Bcl‐2), Bcl‐2‐associated X (BAX), cysteinyl aspartate‐specific proteases‐3 (cas‐3), and cleaved‐caspase‐3 (c‐cas‐3), (AF6416, AF3416, AF6020, AF3020, DF7742, AF0235, AF5128, AF5384, AF4650, AF6139, AF0120, AF6311, AF7022, Affinity Biosciences, Jiangsu, China); Collagen I, Collagen III, alpha‐smooth muscle actin (α‐SMA) (14695‐1‐AP, 22734‐1‐AP, 14395‐1‐AP, Proteintech, Hubei, China); Prestained Protein Marker II (10–200 kDa), Prestained Protein Marker VI (55–320 kDa), (G2058, G2085, Servicebio, Hubei, China); Prestained Protein Ladder (10–180 kDa), (MF212‐01, Mei5Bio, Beijing, China); pECMV‐Foxo1‐m‐FLAG (P6481, Miaoling Plasmid, Hubei, China); si‐FOXO1, NanoTrans 20 (RX26021814067/4068, HPO1001, Hippo bio, Zhejiang, China).

### 2.2 Preparation and HPLC Analysis of APL

The decoction was made at 30 min after soaking in 10 times the volume of crude drug in water. The decoction was filtered and collected. The dregs were again decocted in 8 times the volume of crude drug in water for 30 min and filtered and collected. The two decoctions were mixed, concentrated, and freeze‐dried.

APL was dissolved in methanol (20 mg/mL crude drug). After ultrasonic extraction for 30 min, the solution was filtered through a 0.22 μm microfiltration membrane. HPLC (LC‐2050C 3D, SHIMADZU, Tokyo, Japan) was used to analyze the main active ingredients in APL. The chromatography conditions were as follows: the chromatography column was ShimNex CS C18 (5 μm, 4.6 mm × 250 mm; SHIMADZU, Tokyo, Japan). The column temperature was set at 30°C, the flow rate was 1 mL/min, and the injection volume was 10 μL. Acetonitrile solution was used as mobile phase A, and 0.1% formic acid aqueous solution as mobile phase B. The elution conditions were: 0–30 min, 5%–10% A; 30–50 min, 10%–15% A; 50–80 min, 15%–25% A; 80–90 min, 25%–40% A; 90–105 min, 40%–50% A.

### 2.3 Network Pharmacology Analysis

All structures were acquired from PubChem. The targets of active ingredients in APL were predicted using the TCMSP database (OB ≥ 30%, DL ≥ 0.18), BATMAN‐TCM (score cutoff ≥ 20, *p* < 0.05), and Swiss Target Prediction (probability > 0.1). The targets of *homo sapiens* were obtained from UniProt. Known therapeutic targets of MF and relevant targets of autophagy were obtained from GeneCards (relevance score ≥ 10). The intersecting targets of active ingredients in APL, MF, and autophagy were obtained via Draw Venn Diagram. The protein–protein interaction (PPI) network was constructed using the STRING database (confidence score ≥ 0.4), and core hub genes were identified using CytoHubba in Cytoscape 3.9.1. Metascape was employed for GO and KEGG pathway enrichment analyses (*p* < 0.05). Websites and website guides are shown in Supporting Information.

### 2.4 Animals, Model Establishment and Drug Administration

Male Kunming mice, weighing between 30 and 35 g, were procured from Liaoning Changsheng Biotechnology Co. Ltd., with an approval number of SCXK (Liao) 2020‐0001. The ethical conduct of this study was reviewed and approved by the Ethics Committee of Hebei University of Chinese Medicine, with the certificate number DWLL202403051. These mice were kept in standardized environmental conditions, with free access to food and water.

Mice were divided into six groups through a randomization process: Control group, ISO group (15 mg/kg), PRO group (40 mg/kg), APL‐L group (0.78 g/kg), APL‐M group (1.56 g/kg), and APL‐H group (3.12 g/kg). Except for the Control group, mice in the other groups were injected subcutaneously with ISO, and in the administered groups were given the corresponding drug by gavage. Upon completion of the experiment, the mice were anesthetized with sodium pentobarbital (50 mg/kg) by intraperitoneal injection. Blood samples were collected and centrifugation to isolate serum samples. The hearts were excised, weighed, photographed, and placed in fixative or stored in the refrigerator at −80°C.

### 2.5 Electrocardiography and Echocardiography Analysis

Mice were anesthetized with isoflurane and fixed. Electrocardiography was recorded by the PLC01 PowerLab Collection System (ADInstruments, Shanghai, China) and echocardiography was recorded by the Vevo 2100 Imaging System (Visual Sonics, Toronto, Canada).

### 2.6 Biochemical Index Detection

Blood was centrifuged at 3500 rpm for 15 min at 4°C, and the supernatant was gathered. LDH, CK, and CK‐MB levels were determined using an Automatic Biochemical Analyzer (Icubio, Guangzhou, China). SOD, MDA, GSH‐Px, and CAT levels were determined using an Epoch microplate reader (BioTeK, Vermont, USA).

### 2.7 Enzyme Linked Immunosorbent Assay (ELISA) Detection

The serum TNF, IL‐6, IL‐1β, PICP, and ICTP levels were detected by a Synergy H4 microplate reader BIO‐TEK, USA.

### 2.8 Hematoxylin–Eosin (HE) and Sirius Red Staining

The heart tissue was immobilized, dried, embedded in paraffin, sliced into 5 μm, and deparaffinized. The samples were stained with the HE or Sirius Red dye. Images were captured utilizing a DM4 microscope (LEICA, Germany).

### 2.9 Wheat Germ Agglutinin (WGA) Staining

Following dewaxing to water, tissue sections were inserted in a repair box containing EDTA antigen repair solution for antigen repair. The diluted WGA and DAPI dye solution was added in sequence, and the self‐fluorescence quench agent was added. Photographs and observations were taken of the heart tissue (Hitachi, Japan).

### 2.10 Transmission Electron Microscopy (TEM) Analysis

The tissue was dehydrated, embedded, penetrated by resin, sectioned, and stained. Photographs and observations were taken of the ultrastructure (Hitachi, Japan).

### 2.11 Flow Cytometry

The cardiac tissue was rinsed in PBS, and the cells were collected after digestion. JC‐1 staining solution was added to the cell suspension, mixed adequately, and incubated at 37°C for 20 min. After centrifugation, the cells were resuspended with JC‐1 staining buffer and detected with an Attune NXT Flow cytometer (Thermo, Massachusetts, USA).

### 2.12 Reactive Oxygen Species (ROS) Staining

The frozen sections were shaken dry, ROS staining solution was added dropwise, and incubated for 30 min at 37°C. Following thorough PBS washes, samples were combined with fluorescent secondary antibody. Thereafter, the nuclei were stained with DAPI and visualized using a microscope.

### 2.13 TUNEL Assay

Paraffin sections were deparaffinized, repaired with proteinase K, and equilibrated for 10 min; TUNEL reaction solution was added dropwise and incubated for 1 h at 37°C. After washing with PBS, samples were combined with fluorescent secondary antibody. Thereafter, the nuclei were stained with DAPI and visualized using a microscope.

### 2.14 Immunofluorescence Analysis

The paraffin‐embedded heart tissue was sectioned into 5 μm, deparaffinized, hydrated with xylene and ethanol, and subjected to antigen repaired. Sections were blocked and incubated with COXIV and LC3B antibodies (1:200 dilution). Following thorough PBS washes, samples were combined with fluorescent secondary antibody. Thereafter, the nuclei were stained with DAPI and visualized using a microscope.

### 2.15 Immunohistochemistry Analysis

The paraffin‐embedded heart tissue was sectioned into 5 μm, deparaffinized, hydrated with xylene and ethanol, and antigen repaired. Sections were blocked and incubated with FOXO1A, p‐FOXO1A, FOXO3A, and p‐FOXO3A antibodies (1:200 dilution). Following thorough PBS washes, samples were combined with the second antibody. Thereafter, the nuclei were stained with DAPI and visualized using a microscope.

### 2.16 Cells

H9c2 cells were acquired from Procell (SNL‐029, Hubei, China) and cultivated in DMEM enriched with 10% FBS at 37°C in a 5% CO_2_ environment. Cells were cultured in 96‐well plates (5 × 10^4^ cells/well) for 12 h, then incubated with APL for 24 h. Subsequently, the supernatant was removed and the CCK‐8 kit was added and incubated for 1 h. The absorbance was measured at 450 nm using a VICTOR Nivo microplate reader (PerkinElmer, Germany). H9c2 cells were pretreated with APL for 24 h and treated with 200 μM ISO for 4 h (Wang, Yang, et al. 2023). The cells were divided into Control group, ISO (200 μM) group, APL group (200 μM ISO plus 200 mg/mL APL), pl‐FOXO1 (200 μM ISO, 200 mg/mL APL plus FOXO1 plasmid) group, and pl‐Control group. For FOXO1 knockdown, H9c2 cells were transfected with si‐FOXO1 (or scrambled siRNA as negative control) using Lipofectamine 3000 according to the manufacturer's instructions. After 48 h of transfection, cells were treated with APL and ISO as described above.

### 2.17 Western Blot Analysis

The heart tissue was lysed with RIPA buffer, the protein was extracted and the concentration was determined. 5 × loading buffer was mixed with the protein supernatant in a 1:4 ratio. The mixture was subjected to heat denaturation at 100°C. SDS‐PAGE was conducted to resolve the proteins, and then the protein blot was transferred to a PVDF membrane. The membranes were blocked, followed by overnight incubation at 4°C with GAPDH (1:10000), FOXO1A, p‐FOXO1A, FOXO3A, p‐FOXO3A, PINK1, Parkin, Beclin‐1, p62, LC3B, Collagen I, Collagen III, α‐SMA, BAX, Bcl‐2, caspase‐3, and cleaved‐caspase‐3 (1:1000) antibodies. Following three washes with TBST, the membranes were incubated with a secondary antibody for 1 h. The western blot was visualized by an Intelligent Image Workstation (Biolight Biotechnology, Guangzhou, China).

### 2.18 Statistical Analysis

Results were presented as mean ± SEM and displayed using SPSS 26.0. Data were analyzed by one‐way ANOVA with Tukey's test or Dunnett's T3 test. Data analysis between the two groups was performed using the *t*‐test. *p* < 0.05 was considered statistically significant.

## 3 Results

### 3.1 APL Chemical Ingredients Analyzed by HPLC

**FIGURE 1:**
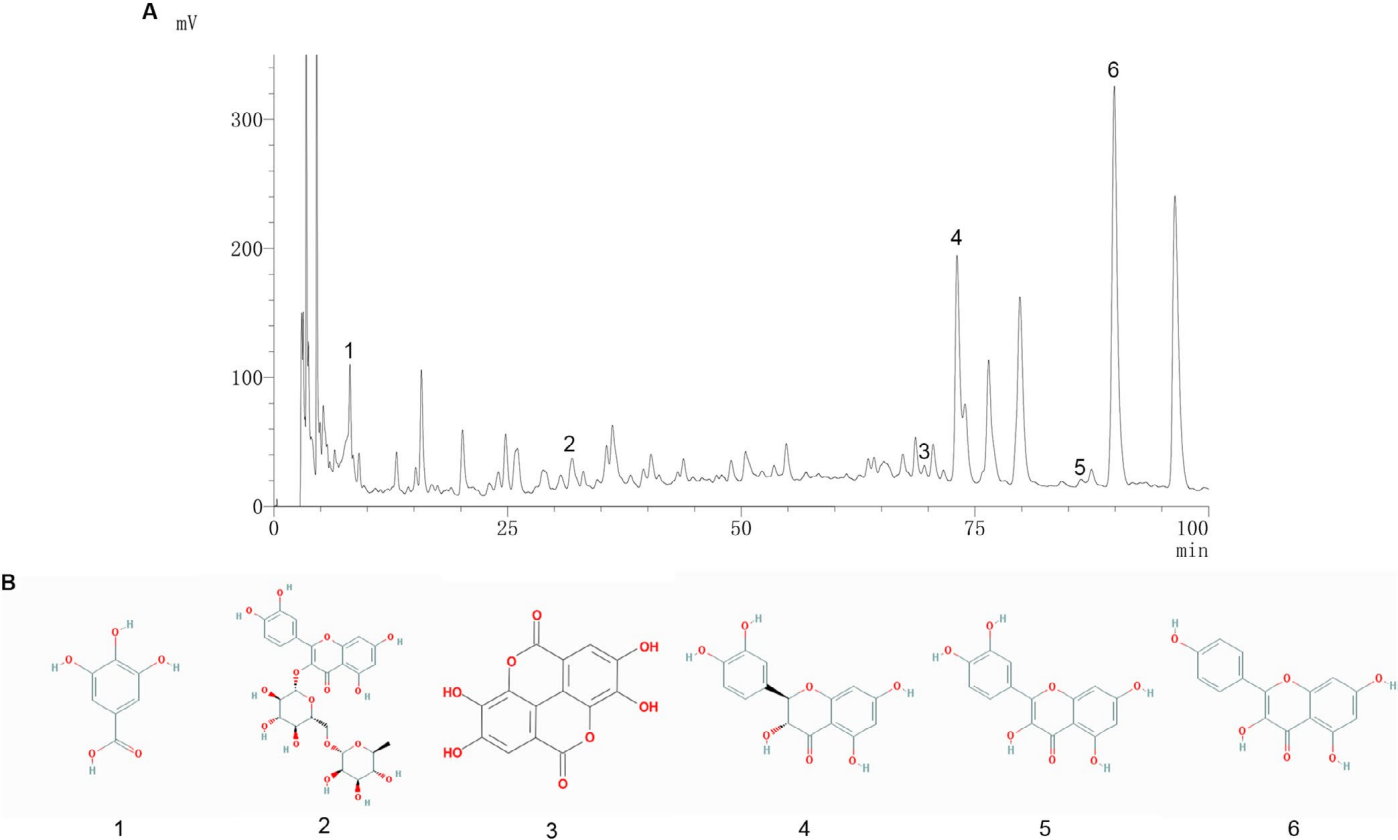
Liquid chromatographic analysis of APL. (A) Standard chromatograms. (B) Chemically active ingredients. 1. Gallic acid, 2. Rutin, 3. Ellagic acid, 4. Taxifolin, 5. Quercetin, 6. Kaempferol.

HPLC analysis indicated that the primary components in APL were Gallic acid, Rutin, Ellagic acid, Taxifolin, Quercetin, and Kaempferol (Figure 1). These ingredients were mainly concentrated in flavonoids and polyphenols.

### 3.2 Network Pharmacology Displayed Relevant Pathways of APL in the Treatment of MF

**FIGURE 2:**
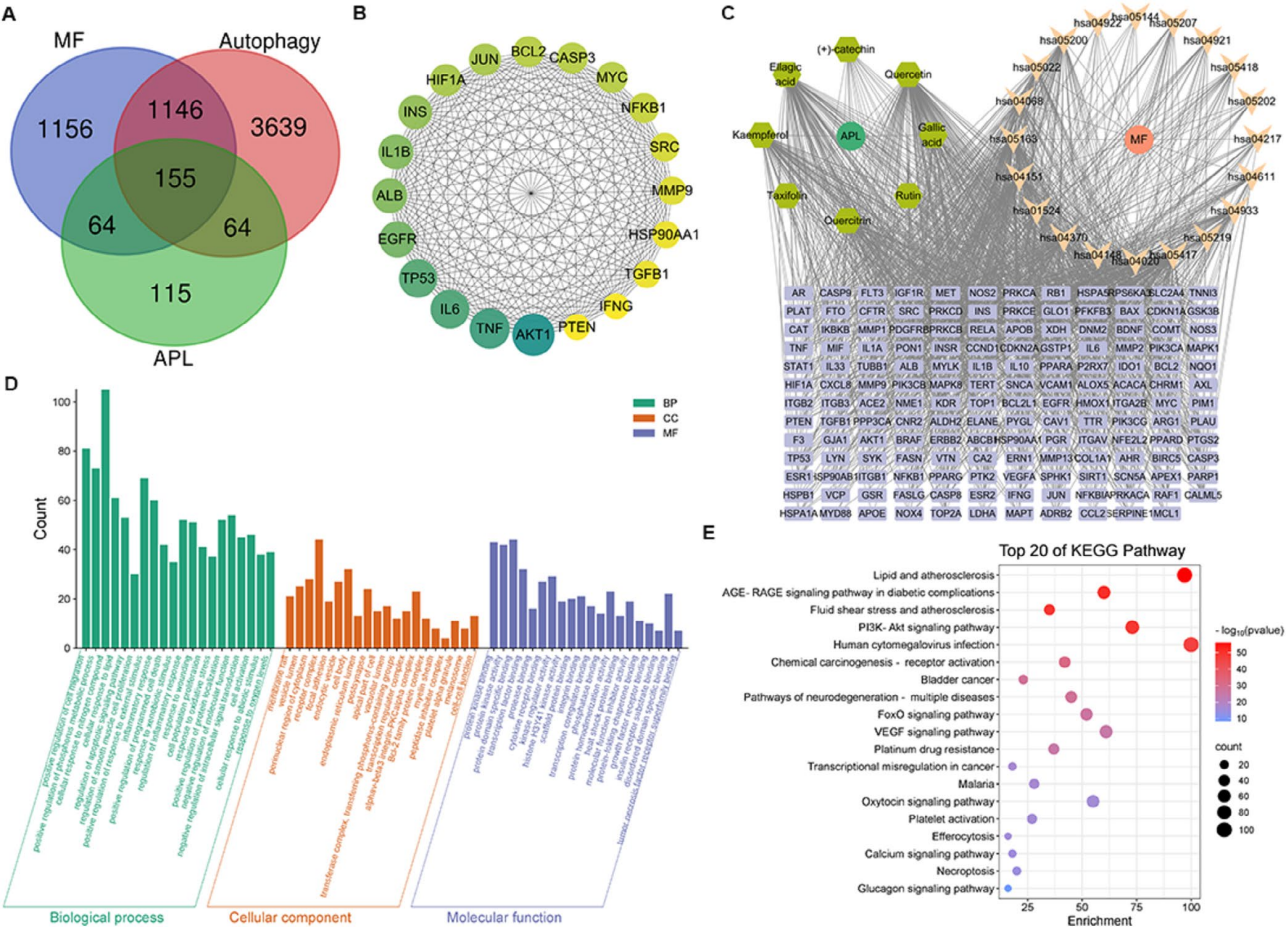
Network pharmacological analysis. (A) Venn diagram of the targets of APL, autophagy, and MF. (B) Top 20 intersecting targets of APL, autophagy, and MF. (C) Drug‐ingredient‐target‐pathway visualization network. (D) GO enrichment analysis. (E) KEGG pathway analysis.

We collected 2521 MF targets, 398 targets of APL, and 5004 autophagy targets for screening. We intersected the MF targets, autophagy targets, and APL targets and identified 155 common targets. Then, we built a PPI network that includes overlapping targets for APL, autophagy, and MF. We identified potential mechanisms for treating MF from an autophagy angle using the KEGG pathway. The 155 intersecting targets were enriched in 184 KEGG pathways, 270 biological processes, 96 cellular components, and 141 molecular functions. KEGG analysis results suggested that the pharmacological mechanisms of APL in treating MF primarily involved the AGE‐RAGE, PI3K‐AKT, and FOXO signaling pathways. GO enrichment analysis results suggested that the biological processes were primarily involved in positive regulation of cell migration, positive regulation of phosphorus metabolic process, and cellular response to nitrogen compound. The cellular components were principally focused on membrane raft, vesicle lumen, and perinuclear region of cytoplasm. The molecular functions mainly related to protein kinase binding, protein kinase activity, and protein domain specific binding (Figure 2).

### 3.3 APL Ameliorated Cardiac Function in MF Mice

**FIGURE 3:**
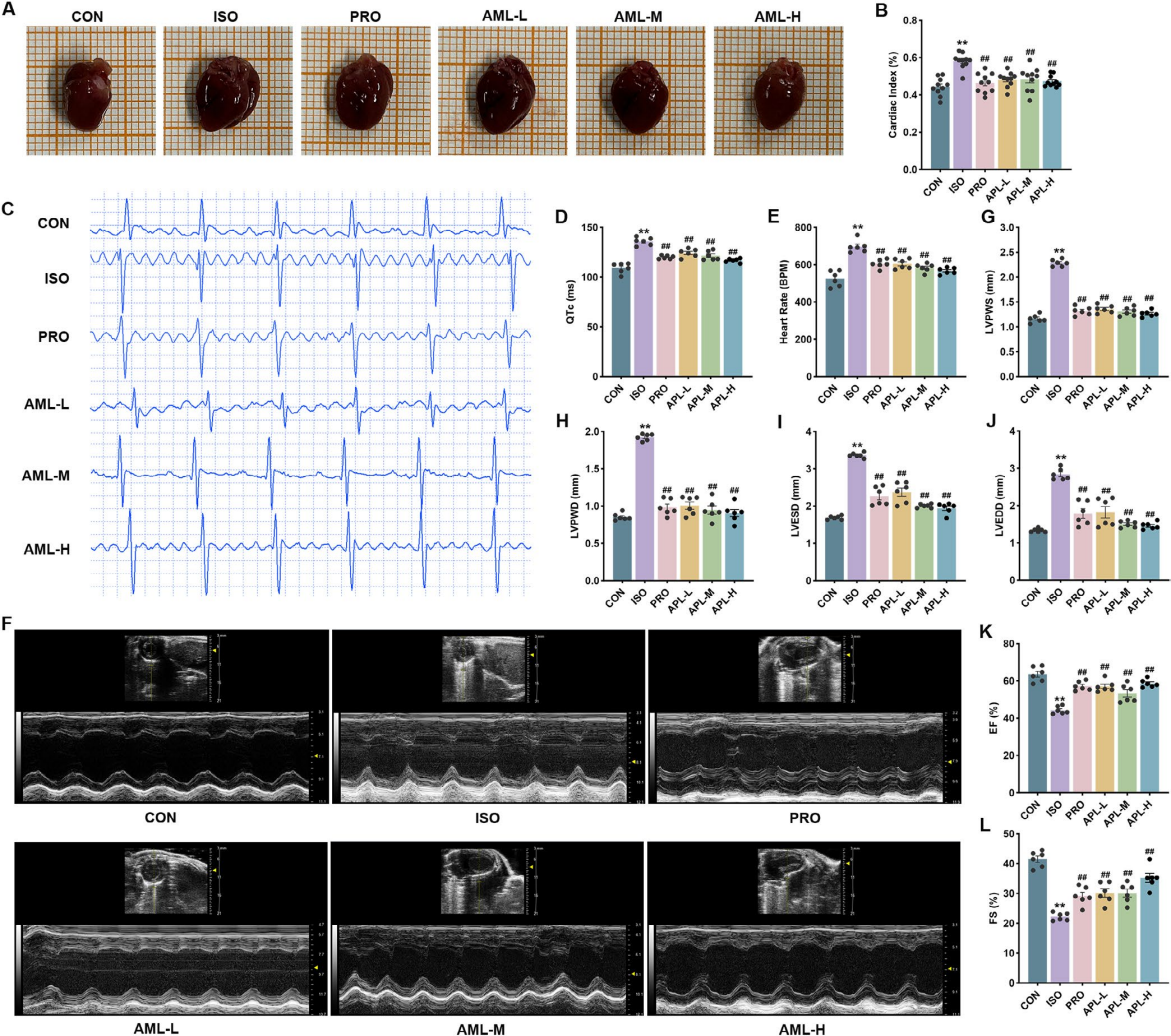
Effects of APL on cardiac function in MF mice. (A) Representative picture of the hearts. (B) Cardiac index of mice. (C) Representative electrocardiogram. (D, E) QTc and heart rate in mice. (F) Representative echocardiography. (G–L) The dynamic changes of LVPWS, LVPWD, LVESD, LVEDD, EF, and FS in mice. Data are presented as mean ± SEM, *n* = 6. **p < 0.01 vs CON group, ^##^ *p* < 0.01 vs ISO group.

The cardiac function of mice was analyzed by electrocardiograph and echocardiography. Cardiac index, QTc, heart rate, LVPWS, LVPWD, LVESD, and LVEDD were increased, while EF and FS were decreased in the ISO group. Compared with the ISO group, cardiac index, QTc, heart rate, LVPWS, LVPWD, LVESD, and LVEDD decreased, while EF and FS increased in each administration group (Figure 3). This indicates that APL possesses a protective effect on cardiac function.

### 3.4 APL Inhibited Myocardial Damage and Inflammation in MF Mice

**FIGURE 4:**
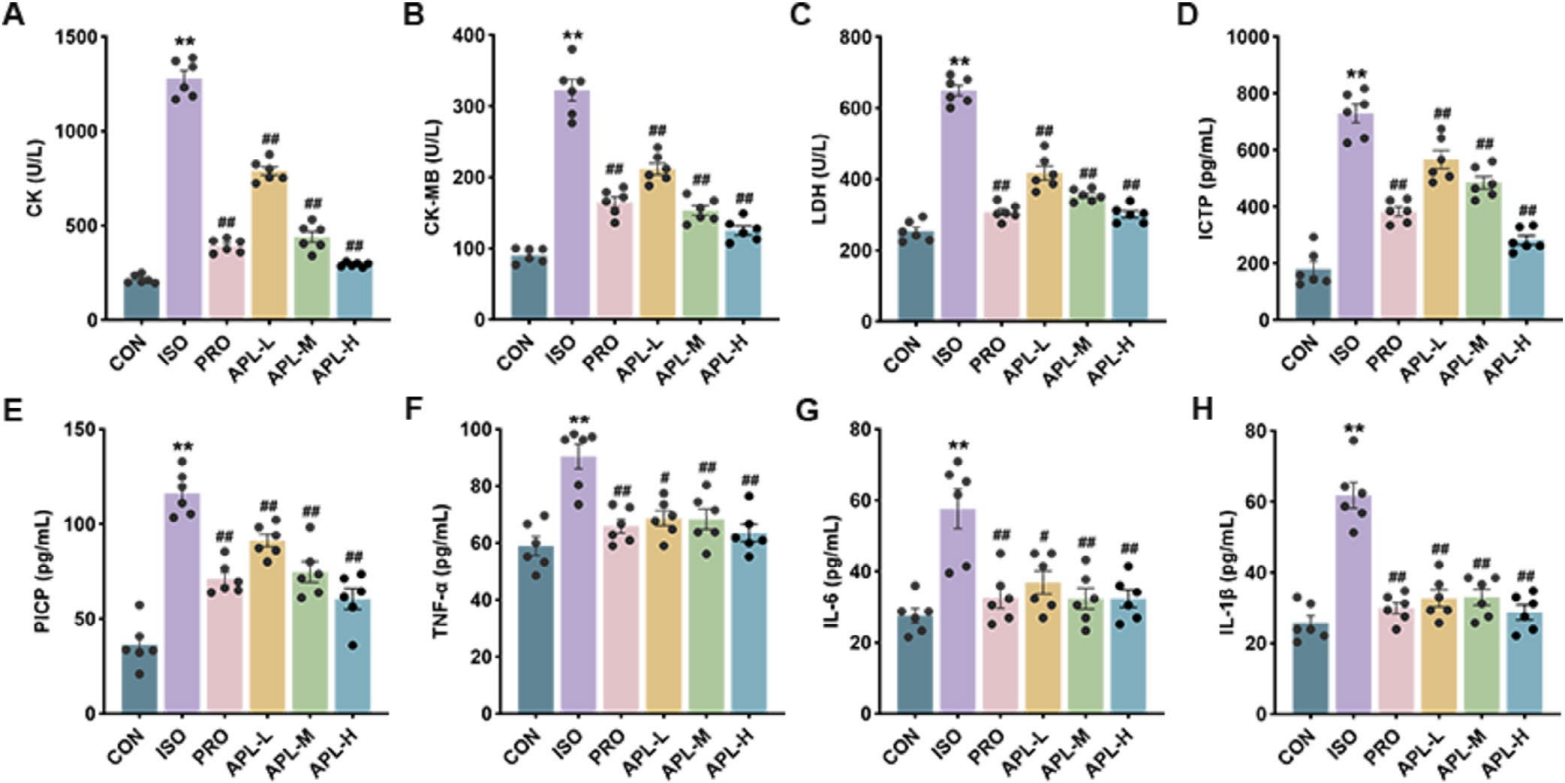
Effects of APL on myocardial injury in mice. (A–H) Levels of CK, CK‐MB, LDH, ICTP, PICP, TNF‐α, IL‐6, and IL‐1β in the serum of mice. Data were displayed as means ± SEM, *n* = 6. **p < 0.01 vs CON group, ^#^ *p* < 0.05, ^##^ *p* < 0.01 vs ISO group.

To investigate the effects of APL on myocardial injury in mice, we examined serum myocardial injury and inflammation indicators. Compared with the CON group, the levels of CK, CK‐MB, LDH, ICTP, PICP, TNF‐α, IL‐6, and IL‐1β increased in the ISO group. After APL and PRO treatment, the levels of the above indicators decreased (Figure 4). Our findings indicate that APL can mitigate myocardial injury, inflammatory response, and fibrosis.

### 3.5 APL Ameliorated Pathological Change and MF

**FIGURE 5:**
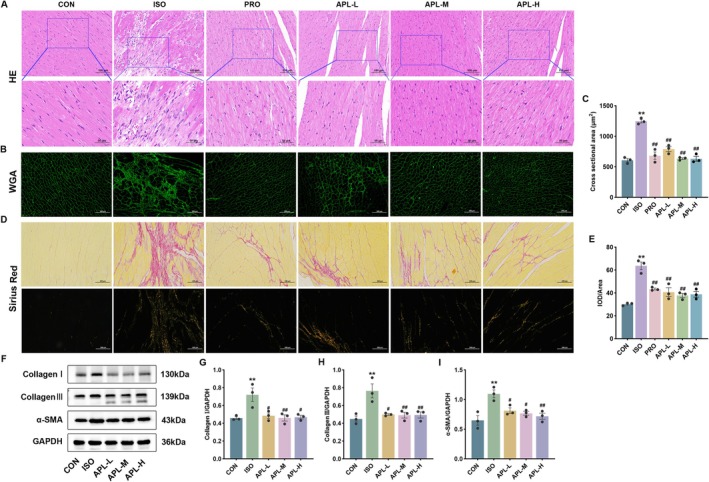
Effects of APL on pathological morphological change and MF in mice. (A) HE staining (200×, 400×). (B, C) WGA staining (200×). (D, E) Sirius Red staining (200×). (F–I) The protein expressions of Collagen I, Collagen III, and α‐SMA. Data were displayed as means ± SEM, *n* = 3. **p < 0.01 vs CON group, ^#^ *p* < 0.05, ^##^ *p* < 0.01 vs ISO group.

HE staining results showed that the cardiomyocytes were structurally intact and tightly aligned in the CON group. Cardiomyocytes were enlarged in size, disorganized, with uneven cytoplasmic staining, accompanied by extensive inflammatory cell infiltration, and some cells were hypertrophic and necrotic in the ISO group. However, APL and PRO interventions improved these pathologic changes Figure 5A.

WGA staining showed that cardiomyocytes of mice in the ISO group were significantly hypertrophied, and APL and PRO could reduce the degree of hypertrophy by reducing cell volume (Figure 5B,C).

Sirius Red staining results indicated that myocardial fibers were arranged clearly and tightly, with very few collagen fibers in the intercellular matrix under normal circumstances. In the ISO group, myocardial fibers were broken and the intercellular matrix was full of collagen fibers. Under the polarized light microscope, orange fluorescence was observed for type I collagen fibers and green fluorescence for type III collagen fibers. Compared with the ISO group, the myocardial collagen fibers were reduced in the myocardial tissue of mice in APL and PRO groups (Figure 5D,E). Similarly, in the ISO group, western blotting results showed that Collagen I, Collagen III, and α‐SMA expressions were increased and decreased after APL treatment (Figure 5F–I). These findings suggest that APL can mitigate myocardial tissue injury, necrosis, and inflammatory cell infiltration, while improving myocardial cell hypertrophy and fibrosis.

### 3.6 APL Alleviated Apoptosis and Oxidative Stress in MF Mice

**FIGURE 6:**
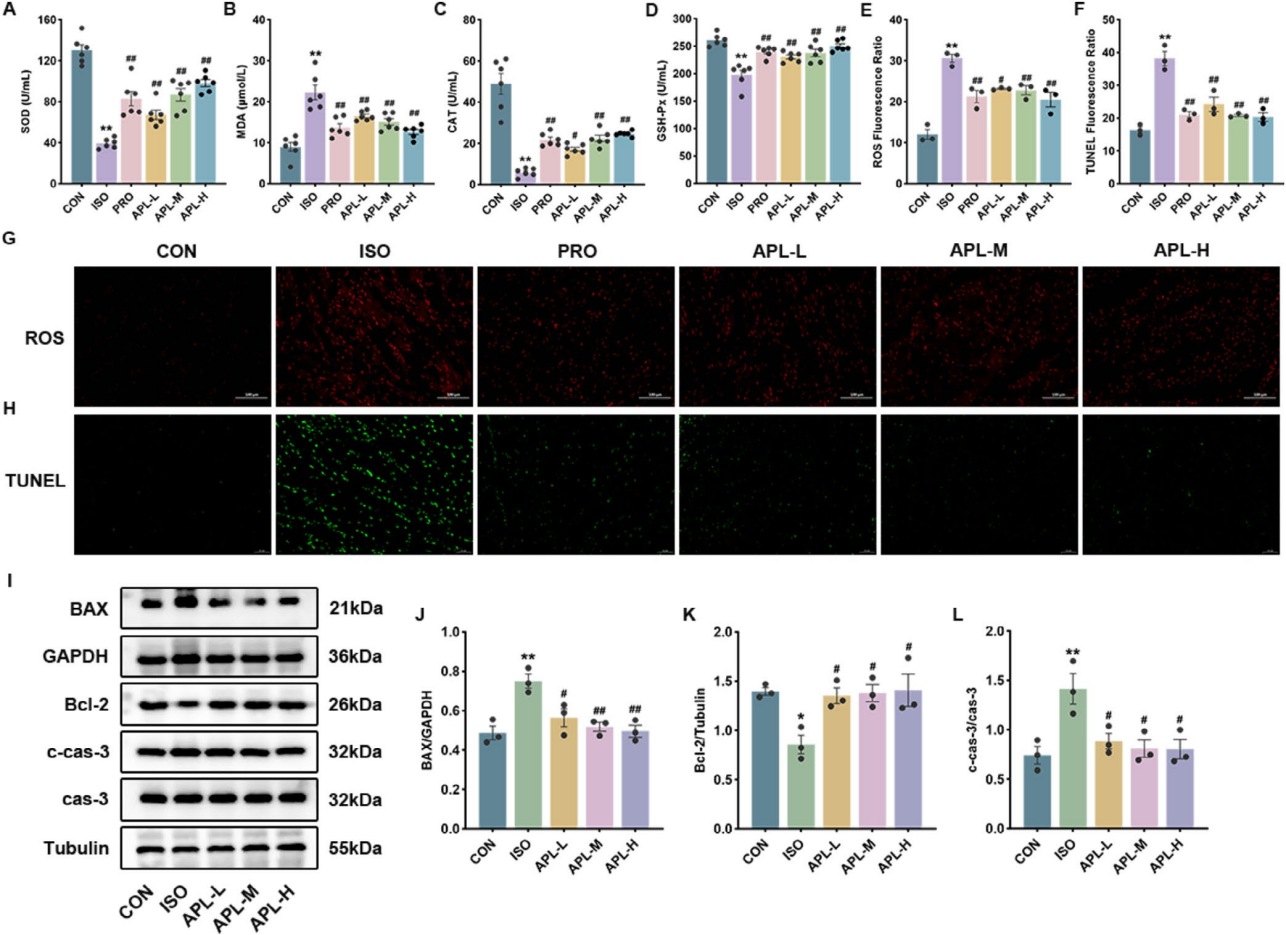
Effects of APL on apoptosis in mice. (A–D) Levels of SOD, MDA, CAT, and GSH‐Px in the serum of mice. (E, G) Levels of ROS in the heart of mice (200×). (F, H) Apoptosis rate in the heart of mice (200×). (I–L) The protein expressions of BAX, Bcl‐2, and c‐cas‐3. Data were displayed as means ± SEM, *n* = 3 or 6. *p < 0.05, **p < 0.01 vs CON group, ^#^ *p* < 0.05, ^##^ *p* < 0.01 vs ISO group.

By examining representative indicators of oxidative stress, we found a significant increase in serum MDA levels and ROS content in the heart, and a significant decrease in serum SOD, CAT and GSH‐Px levels in the ISO group. All the above conditions were improved after APL treatment. By detecting apoptosis‐related indexes, we found that the apoptosis rate and the levels of apoptosis‐related proteins BAX and c‐cas‐3 were up‐regulated, and the expression of Bcl‐2 was down‐regulated in the hearts of the ISO group. APL could significantly reduce the apoptosis rate, decrease BAX and c‐cas‐3 expressions, and increase Bcl‐2 expression (Figure 6). These results indicate that APL can alleviate oxidative stress and apoptosis in myocardial tissue.

### 3.7 APL Alleviated Mitochondrial Function and Mitophagy

**FIGURE 7:**
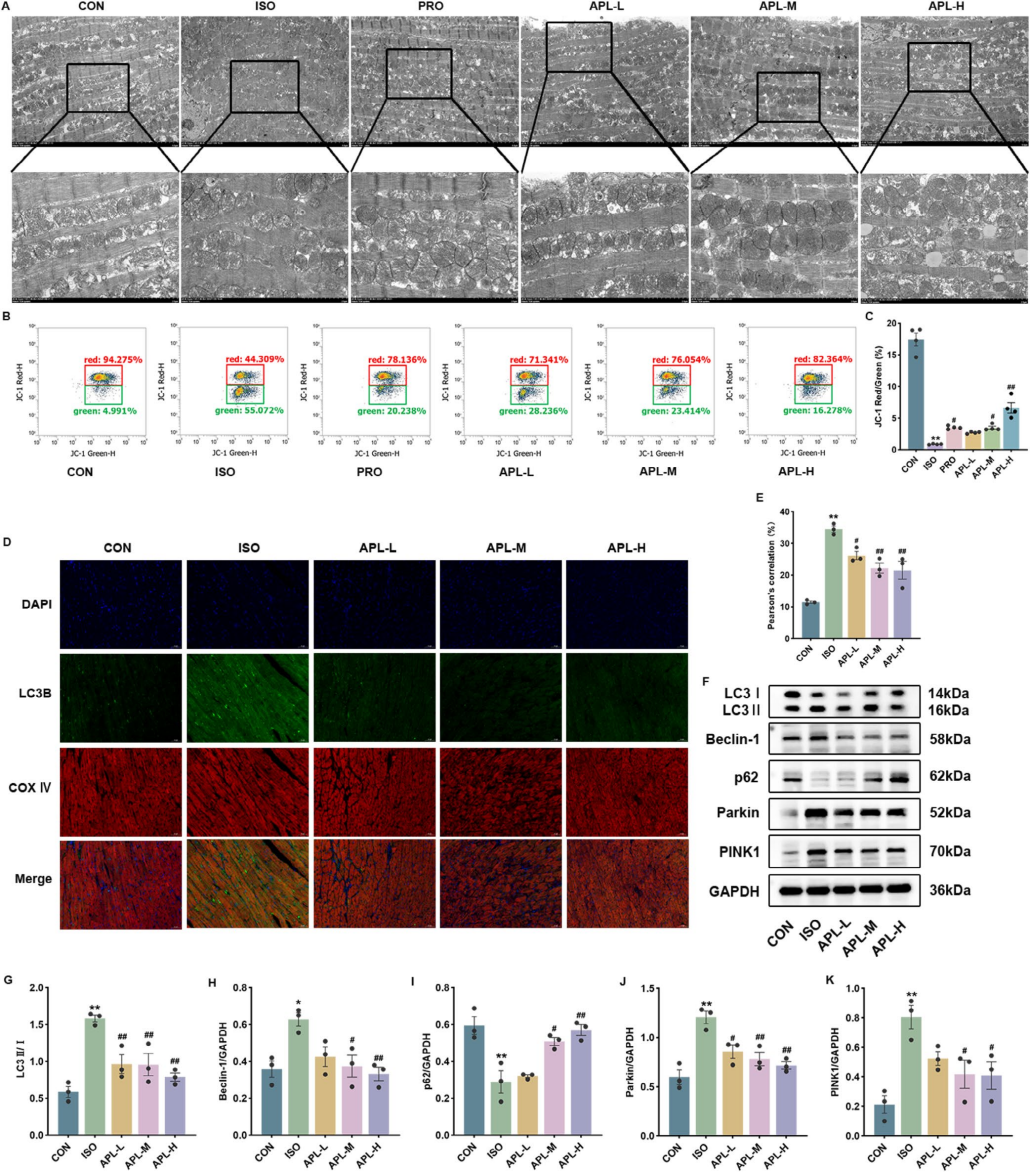
Effects of APL on mitochondrial function and mitophagy of MF mice. (A) Images of heart tissue ultrastructure. (B, C) Mitochondrial membrane potential. (D, E) Images of co‐localization of COX IV and LC3B (×200). (F–K) The protein expressions of LC3II, p62, Beclin‐1, PINK1, and Parkin. Data were displayed as means ± SEM, *n* = 3 or 4. *p < 0.05, **p < 0.01 vs CON group, ^#^ *p* < 0.05, ^##^ *p* < 0.01 vs ISO group.

TEM was used to examine the mitochondrial structure and mitophagy in each group of mice. After ISO intervention, the mitochondrial structure of cardiomyocytes was damaged and the presence of mitochondrial autophagosomes was observed. However, the mitochondrial structure was restored and mitochondrial autophagosomes were reduced in the APL group (Figure 7A).

Flow cytometry was used to detect the mitochondrial membrane potential (MMP), and the results indicated that the MMP of cardiomyocytes of mice in the ISO group was significantly declined, and the MMP was significantly elevated in each of the administered groups (Figure 7B,C).

Mitochondria and autophagosomes were labeled using COX IV and LC3B double staining, respectively. Co‐localization of COX IV and LC3B was observed under a confocal microscope to assess mitophagy levels. The expression of LC3B was increased and co‐localization of LC3B and COX IV was enhanced in the heart tissue in MF mice. Nevertheless, the expression of LC3B was reduced, and co‐localization of LC3B and COX IV was attenuated in the APL group (Figure 7D,E). Subsequently, we assessed the effects of APL on mitophagy using western blotting analysis. LC3II, Beclin‐1, PINK1, and Parkin protein expression levels were up‐regulated and p62 protein expression level was down‐regulated in the ISO group. Conversely, with APL treatment, there was a down‐regulation of protein levels for LC3II, Beclin‐1, PINK1, and Parkin, and an up‐regulation of p62 protein expression level (Figure 7F–K). The results above indicate that APL can restore mitochondrial structure, protect mitochondrial function, and suppress mitophagy.

### 3.8 APL Regulated the FOXO Signaling Pathway in MF Mice

**FIGURE 8:**
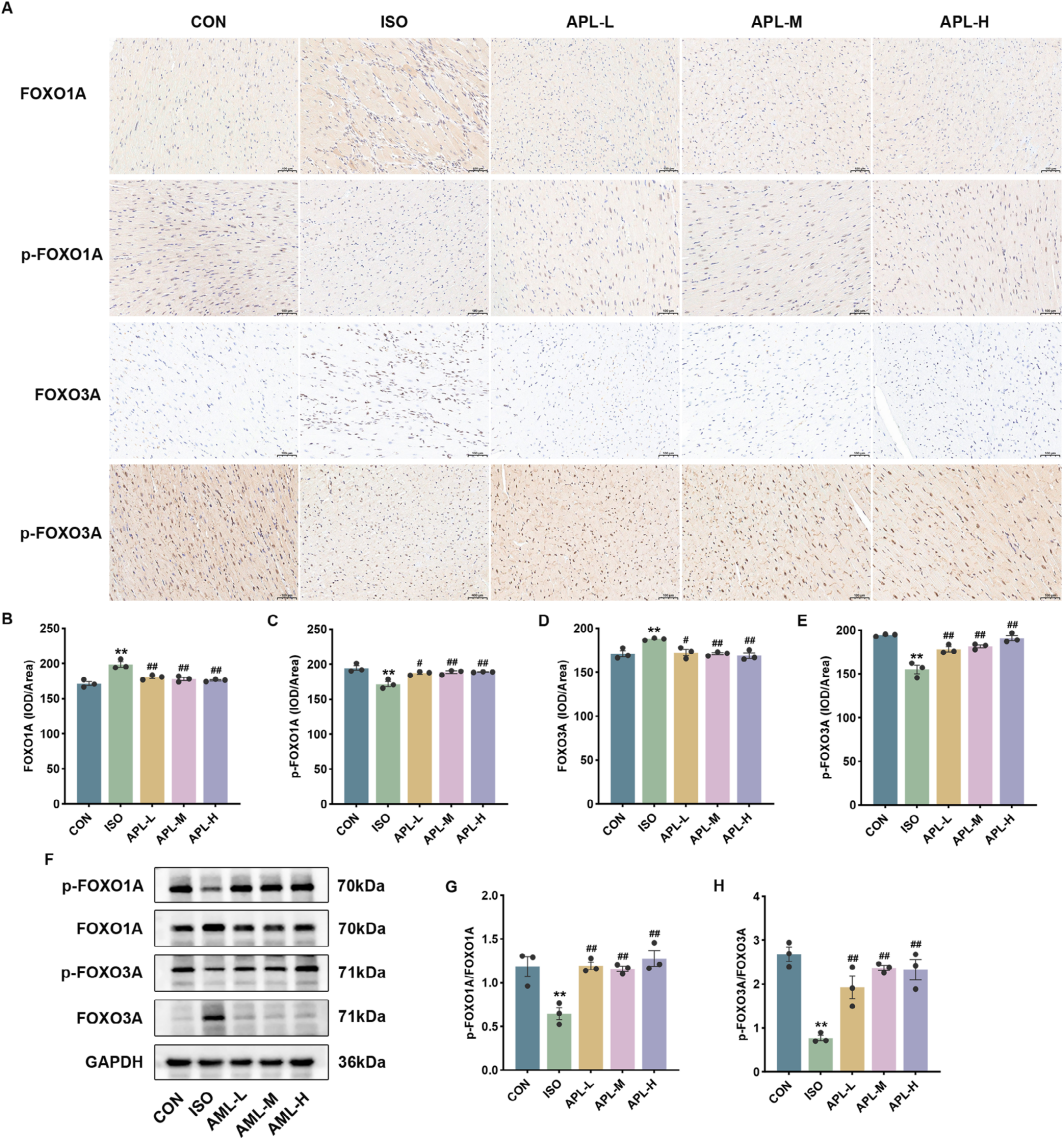
Effects of APL on the FOXO signaling pathway in mice. (A–E) The expressions of FOXO1A, FOXO3A, p‐FOXO1A, and p‐FOXO3A in the heart of mice (immunohistochemical method, 200×). (F–H) The expressions of FOXO1A, FOXO3A, p‐FOXO1A, and p‐FOXO3A in the heart of mice (western blotting). Data were displayed as means ± SEM, *n* = 3. **p < 0.01 vs CON group, ^#^ *p* < 0.05, ^##^ *p* < 0.01 vs ISO group.

Based on network pharmacology results and references, we chose the FOXO signaling pathway for the next study. FOXO1A and FOXO3A expressions were significantly increased and p‐FOXO1A and p‐FOXO3A expressions were significantly decreased in MF mice. Notably, increased nuclear localization of FOXO1A and FOXO3A was observed in the ISO group. The intervention of APL reversed the expression levels of FOXO1A, FOXO3A, p‐FOXO1A, and p‐FOXO3A (Figure 8). Our results indicate that APL can regulate the protein localization, expression, and phosphorylation of FOXO1A and FOXO3A.

### 3.9 APL Inhibited the FOXO1‐Mediated Mitophagy and Fibrosis in H9c2 Cells

**FIGURE 9:**
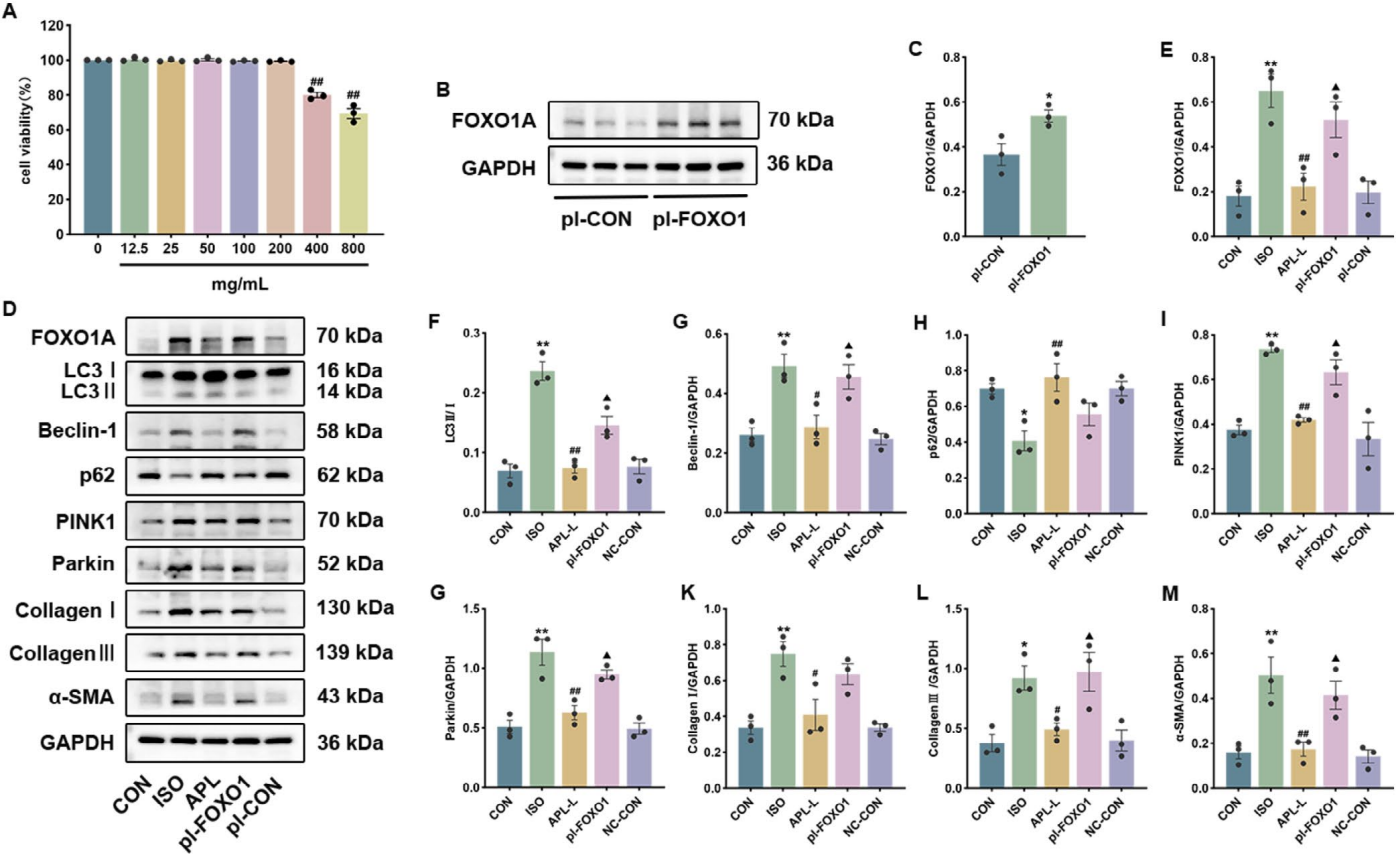
Effects of APL on cell viability, mitophagy, and FOXO1 signaling pathway in H9c2 cells. (A) The cytotoxicity of APL concentrations on H9c2 cells was detected by CCK‐8. (B, C) The protein expressions of FOXO1A following plasmid transfection in H9c2 cells. (D‐M) The protein expressions of FOXO1A, LC3, Beclin‐1, p62, PINK1, Parkin, Collagen I, Collagen III, and α‐SMA were assessed. Data were displayed as means ± SEM *n* = 3. *p < 0.05, **p < 0.01 vs CON group, ^#^ *p* < 0.05, ^##^ *p* < 0.01 vs ISO group, ^▲^ *p* < 0.05 vs pl‐FOXO1 group.

The cytotoxicity of APL on H9c2 cells was first evaluated using the CCK‐8 assay across a range of concentrations (0–800 mg/mL). As shown in Figure 9A, APL at 200 mg/mL showed no significant cytotoxicity while maintaining protective activity; thus, this concentration was selected for subsequent in vitro experiments.

We overexpressed FOXO1 in H9c2 cells and detected the expression of FOXO1 and mitophagy and fibrosis‐related proteins. The protein levels of FOXO1A, LC3B, Beclin‐1, PINK1, Parkin, Collagen I, Collagen III, and α‐SMA were found to be up‐regulated. Meanwhile, the p62 protein expression level was down‐regulated in the ISO group. However, with APL treatment, the protein expression levels of FOXO1A, LC3B, Beclin‐1, PINK1, Parkin, Collagen I, Collagen III, and α‐SMA were down‐regulated, while p62 protein expression level increased (Figure 9). In vitro experiments suggest that APL alleviates MF by modulating mitophagy, an effect mediated through the inhibition of the FOXO signaling pathway.

### 3.10 FOXO1 Is the Critical Target for APL to Regulate Mitophagy and Improve MF

**FIGURE 10:**
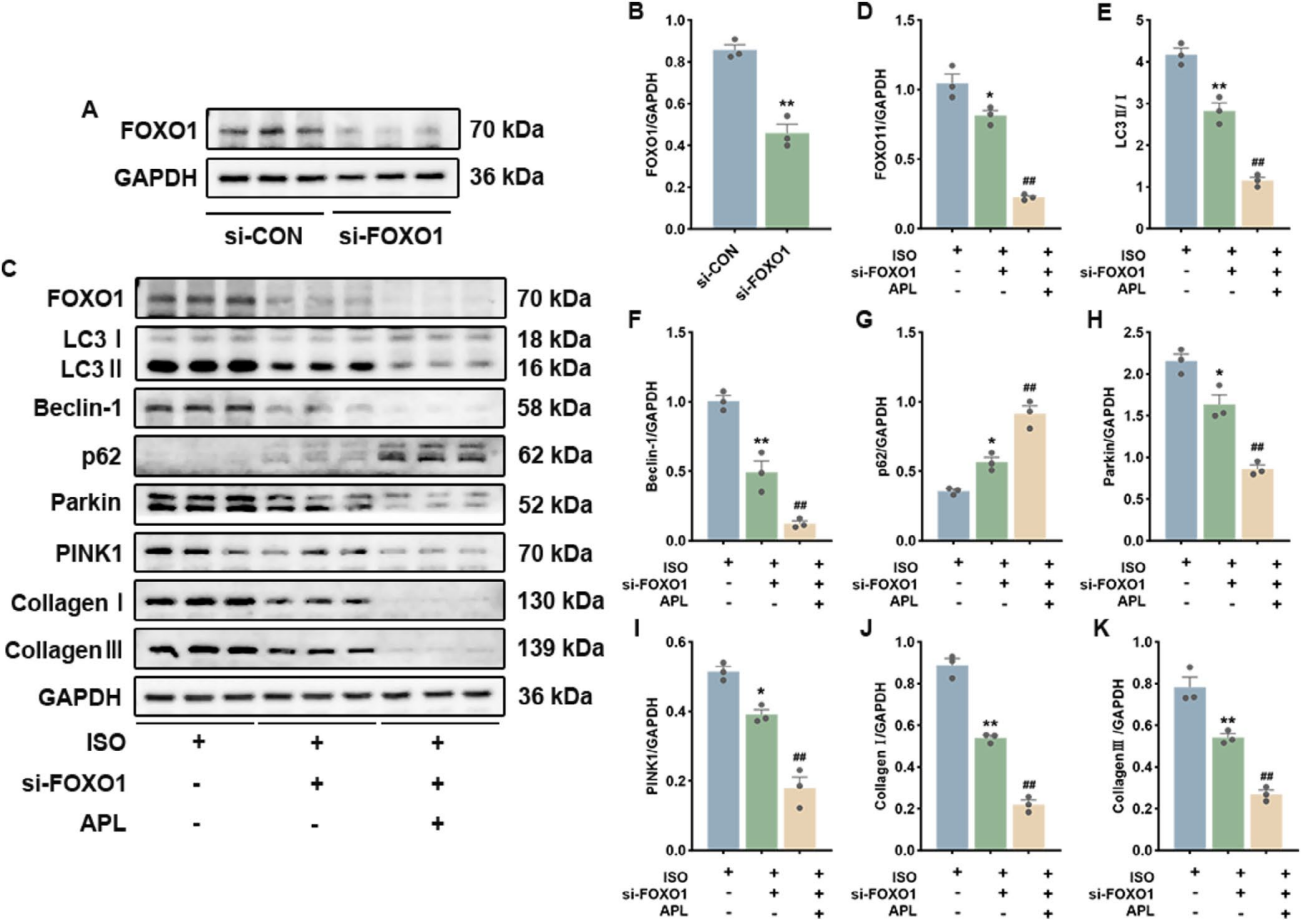
Effects of FOXO1 knockdown on the mitophagy and fibrosis in H9c2 cells. (A, B) The transfection efficiency of si‐FOXO1 (*n* = 3). (C–K) Western blotting results and relative protein expression levels of FOXO1, LC3, Beclin‐1, p62, PINK1, Parkin, Collagen I, and Collagen III (*n* = 3). All values are expressed as mean ± SEM. *p < 0.05, **p < 0.01 vs ISO + si‐FOXO1 group, ^##^ *p* < 0.01 vs ISO + si‐FOXO1 + APL group.

To further validate the role of FOXO1 in mediating the effects of APL, we performed FOXO1 knockdown in ISO‐induced H9c2 cells. As shown in Figure 10A,B, FOXO1 protein expression was significantly reduced in the si‐FOXO1 group, confirming efficient knockdown. In ISO‐induced cells, knockdown of FOXO1 significantly inhibited mitophagy and improved fibrosis. When FOXO1 was inhibited, APL treatment further promoted these changes (Figure 10C–K). These results indicate that FOXO1 is the critical target for APL to regulate mitophagy and improve MF.

## 4 Discussion

The core mechanism of MF involves an abnormal repair response following myocardial injury, characterized by immune and inflammatory dysregulation, non‐cardiac cell proliferation, and scar formation (Guo et al. 2025; Niu et al. 2025). Upon myocardial injury, resident cardiac fibroblasts are activated and differentiate into mature myofibroblasts. These activated myofibroblasts excessively secrete extracellular matrix components, primarily type I and type III collagen, along with various glycoproteins. Ultimately, the scar tissue matures and replaces the original damaged lesion in situ (Yücel et al. 2025). APL is a natural plant whose ingredients are known to protect cardiomyocytes from apoptosis, inhibit oxidative stress, and improve mitochondrial function (Wang et al. 2022). The previous study has indicated that APL can treat AMI by reducing apoptosis and oxidative stress (Zhang, Chen, et al. 2022). Nevertheless, the effect of APL on MF and its specific molecular mechanisms remain to be elucidated.

This study employed a combination of in vivo and in vitro experiments, HPLC analysis, and network pharmacology to systematically evaluate the cardioprotective effects of APL and investigate its mechanism against MF. The results demonstrate that APL significantly mitigates ISO‐induced cardiac injury. Mechanistic studies revealed that the cardioprotective effect is mediated through the regulation of antioxidant and anti‐inflammatory responses, alongside the modulation of FOXO and mitophagy‐related signaling pathways. These findings provide a solid scientific basis for developing APL as a potential cardioprotective functional food or adjunctive formulation.

Electrocardiography and echocardiography are widely used techniques for evaluating the structure and function of the heart. CK, CK‐MB, and LDH levels are essential parameters in the assessment of cardiac injury. In our study, after ISO intervention, QTc, heart rate, LVPWS, LVPWD, LVESD, and LVEDD were increased, while EF and FS were decreased. Previous studies have shown that ISO can induce pathological changes such as myocardial injury, hypertrophy, and fibrosis (Peng et al. 2024). In this study, we found hypertrophy of cardiomyocytes, accompanied by infiltration of inflammatory cells and deposition of large amounts of collagen fibers in the interstitial spaces after subcutaneous injection of ISO in mice. Subsequently, injured cardiomyocytes released numerous cardiac enzymes, resulting in the elevation of serum levels of CK, CK‐MB, and LDH. However, the above pathologic manifestations, cardiac function, and cardiac enzyme levels were reversed after APL administration. Importantly, fibrosis markers such as ICTP and PICP concentrations as well as representative proteins such as Collagen I, Collagen III, and α‐SMA expressions were significantly inhibited, demonstrating that APL protected cardiac function and alleviated myocardial damage and fibrosis.

Previous studies have shown that MF or injury is usually accompanied by oxidative stress and cardiomyocyte apoptosis (Zhang, Li, et al. 2022). Oxidative stress is defined by the excessive generation of ROS (Yan et al. 2018). The final metabolite of ROS is MDA, whose increased concentration can damage cells. Various antioxidative enzymes contribute to ROS elimination through diverse biological pathways and mechanisms, thus antioxidant enzymes such as SOD, CAT, and GSH‐Px were measured to reflect the antioxidant activity of the organism. Moreover, the accumulation of ROS can cause mitochondrial dysfunction, reduce regulated mitochondrial metabolism, and promote leakage of pro‐apoptotic factors, leading to apoptosis (Aparicio et al. 2018). During apoptosis, Bcl‐2, as an anti‐apoptotic protein, binds to the pro‐apoptotic protein BAX to promote the release of apoptosis‐inducing factors, which in turn induce apoptosis in a cascade reaction with caspase family proteins. Notably, the expression of c‐cas‐3 after activation is considered to be a hallmark of apoptosis. Studies have shown that ISO can induce oxidative stress and cardiomyocyte apoptosis (Yang et al. 2022). After ISO induction, ROS, MDA, BAX, and c‐cas‐3 levels increased, whereas SOD, CAT, and GSH‐PX activities decreased, resulting in oxidative stress and apoptosis in myocardial tissue. This phenomenon can be reversed by intervention with APL, suggesting that APL has antioxidant and anti‐apoptotic effects, which is consistent with a previous study (Zhang, Chen, et al. 2022).

The main ingredients in APL were analyzed by HPLC, and the studies showed that all of these ingredients including Gallic acid (Yan et al. 2019), Rutin (Huang et al. 2017), Ellagic acid (Wei et al. 2017), Taxifolin (H. Guo et al. 2015), Quercetin (Albadrani et al. 2021), and Kaempferol (Du et al. 2019) had the effect of reducing MF. The synergy of multiple active ingredients optimized the anti‐MF efficacy of APL. Therefore, in the network pharmacology study, we used the above‐mentioned ingredients for analysis. Network pharmacological analyses indicated that APL may improve MF by modulating AGE‐RAGE, PI3K‐AKT, and FOXO signaling pathways. These results deepen our understanding of the anti‐MF effects of APL and its bioactive ingredients.

FOXO transcription factors are a group of highly conserved proteins, including FOXO1, FOXO3, FOXO4, and FOXO6, whose functions are regulated mainly by post‐translational modifications such as phosphorylation (Eijkelenboom and Burgering 2013). The phosphorylated FOXO will be degraded, and the undegraded FOXO will enter the nucleus and regulate the transcriptional activity of downstream genes, thereby modulating a series of biological processes, including autophagy, apoptosis, oxidative damage, and inflammatory responses (Jin and Hou 2024). The studies suggested that FOXO1 and FOXO3 could regulate mitophagy and attenuate MF (Zhang et al. 2021; Zhou et al. 2024). The present investigation indicated that APL diminished the up‐regulation of FOXO1A and FOXO3A proteins as well as the down‐regulation of p‐FOXO1A and p‐FOXO3A proteins triggered by ISO. Significantly, the nuclear localization of FOXO1A and FOXO3A was increased in the MF mice heart, which was reversed by APL intervention.

FOXO3 usually accompanies FOXO1 into the nucleus, but can also translocate to mitochondria to exert antioxidant effects and regulate mitophagy (Fasano et al. 2019). Mitophagy is a cellular mechanism to specifically remove impaired mitochondria through self‐degradation and maintain cellular physiological functions. However, overactivation of mitophagy removes large numbers of mitochondria from the cell, leading to impaired ATP synthesis, which affects cardiac contractile function (Jimenez et al. 2014). LC3B, Beclin‐1, and p62 are autophagy core proteins that are used to evaluate autophagy levels. COXIV is a mitochondrial endosomal membrane marker that is usually assayed for co‐localization with LC3B to evaluate mitophagy levels. Crucially, the PINK1/Parkin pathway is instrumental in mediating the clearance of dysfunctional mitochondria through mitophagy. When mitochondria are damaged, the inner mitochondrial membrane undergoes depolarization. At this time, PINK1 accumulates on the outer mitochondrial membrane and recruits Parkin, activating mitophagy (Kong et al. 2024). It has been found that inhibition of PINK1 and Parkin expression attenuated excessive mitophagy and protected cardiomyocytes from damage (Guo et al. 2024). Similarly, after ISO intervention, the loss of MMP and an increase in PINK1/Parkin‐mediated mitophagy were detected. In contrast, APL attenuated mitochondrial damage in cardiomyocytes, restored the damaged MMP, and inhibited mitophagy. Subsequently, we artificially elevated FOXO1 expression in vitro, which reversed the effects of APL, as evidenced by increased expression of mitophagy and fibrosis‐related proteins. By artificially inhibiting the expression of FOXO1, we demonstrated that FOXO1 is a key target for regulating mitophagy and improving MF.

However, this study still has some limitations. Since we did not perform experiments with individual compounds, the direct link between specific active ingredients and the observed anti‐MF effects remains unclear. Future studies are warranted to clarify the material basis of APL and explore the potential synergistic actions among its constituents. We acknowledge that further studies, including active ingredient verification, validation in additional MF models, and preliminary pharmacokinetic analysis, are warranted to fully establish the translational potential of APL. These will be addressed in our future investigations.

## 5 Conclusions

In summary, APL has the effect of repairing myocardial injury, reducing myocardial hypertrophy, and improving MF. Mechanistically, APL can inhibit mitophagy mediated by the FOXO signaling pathway in vitro and in vivo. This investigation offers foundational evidence for employing APL in MF therapy, highlighting its extensive prospects as a functional food or adjunctive therapeutic agent.

## Author Contributions

**Jian Chen:** software, methodology, data curation, writing – original draft. **Zhixiang Wei:** writing – original draft, formal analysis, data curation, resources. **Jiaqi An:** software, methodology, data curation. **Dantong Li:** formal analysis, methodology. **Ying Gu:** resources, methodology. **Yixin Zhang:** writing – review and editing, Supervision, Project administration. **Muqing Zhang:** writing – review and editing, funding acquisition, supervision.

## Funding

This work was supported by the Foundation of Administration of Traditional Chinese Medicine of Hebei Province (No. 2021051).

## Ethics Statement

The ethical conduct of this study was reviewed and approved by the Ethics Committee of Hebei University of Chinese Medicine, with the certificate number DWLL202403051.

## Conflicts of Interest

The authors declare no conflicts of interest.

## Supporting information


**Table S1:** Websites and website guides.

## Data Availability

The data that support the findings of this study are available from the corresponding author upon reasonable request.
