# Supplementary material for: Protective Effects of Agrimonia pilosa Ledeb. In Myocardial Fibrosis: Inhibition of Mitophagy Mediated by the FOXO Signaling Pathway
Source: Food Sci Nutr. 2026 Apr 15;14(4):e71778. doi: 10.1002/fsn3.71778 (PMC13082918; doi:10.1002/fsn3.71778)
Supplement: Supplementary file 1 — Table S1: Websites and website guides. [file FSN3-14-e71778-s001.docx]

Table S1 Websites and website guides.

| **website** | **website guide** |
| --- | --- |
| PubChem | https://pubchem.ncbi.nlm.nih.gov/ |
| TCMSP database | https://www.tcmsp-e.com/ |
| BATMAN | http://bionet.ncpsb.org.cn/batman-tcm/index.php/ |
| [SwissTargetPrediction](http://www.swisstargetprediction.ch/) | http://www.swisstargetprediction.ch/ |
| UniProt | https://www.uniprot.org/ |
| GeneCards | https://www.genecards.org/ |
| Draw Venn Diagram | https://bioinformatics.psb.ugent.be/webtools/Venn/ |
| STRING database | https://string-db.org/ |
| Metascape | https://metascape.org/gp/index.html#/ |
